# Mutational analysis of the *PITX2 *coding region revealed no common cause for transposition of the great arteries (dTGA)

**DOI:** 10.1186/1471-2350-6-20

**Published:** 2005-05-12

**Authors:** Nadja Muncke, Beate Niesler, Ralph Roeth, Karin Schön, Heinz-Juergen Rüdiger, Elizabeth Goldmuntz, Judith Goodship, Gudrun Rappold

**Affiliations:** 1 Institut für Humangenetik, Universität Heidelberg, INF 366, 69120 Heidelberg, Germany; 2 Abteilung für Kardiologie, Kinderklinik Heidelberg, INF 153, 69120 Heidelberg, Germany; 3 Division of Cardiology, Department of Pediatrics, University of Pennsylvania, School of Medicine, Philadelphia, Pennsylvania 19104, USA; 4 Institute of Human Genetics, International Center for Life, Central Parkway, Newcastle upon Tyne, NE1 3BZ, UK

## Abstract

**Background:**

PITX2 is a bicoid-related homeodomain transcription factor that plays an important role in asymmetric cardiogenesis. Loss of function experiments in mice cause severe heart malformations, including transposition of the great arteries (TGA). TGA accounts for 5–7% of all congenital heart diseases affecting 0.2 per 1000 live births, thereby representing the most frequent cyanotic heart defect diagnosed in the neonatal period.

**Methods:**

To address whether altered PITX2 function could also contribute to the formation of dTGA in humans, we screened 96 patients with dTGA by means of dHPLC and direct sequencing for mutations within the *PITX2 *gene.

**Results:**

Several SNPs could be detected, but no stop or frame shift mutation. In particular, we found seven intronic and UTR variants, two silent mutations and two polymorphisms within the coding region.

**Conclusion:**

As most sequence variants were also found in controls we conclude that mutations in *PITX2 *are not a common cause of dTGA.

## Background

With a frequency of up to 1%, congenital heart disease represents one of the most common major congenital anomalies [1-3]. Transposition of the great arteries (TGA) accounts for 5% of all congenital heart defects [4]. TGA manifests during the early fifth week of development affecting the septation of the common outflow tract into aorta and pulmonary arteries, and has been suggested to represent a laterality defect of the heart [5]. The more common dTGA (dextro-looped TGA) represents a complete inversion of the great vessels (atrioventricular concordance and ventriculoarterial discordance). In the less common lTGA (laevo-looped TGA), both atrioventricular and ventriculoarterial discordance is present. Despite the high prevalence and clinical importance of TGA, we are just beginning to unravel the etiology of this heterogeneous disease. Up to now, three genes have been suggested to be involved in the etiology of dTGA in humans: *PROSIT240*, a novel *TRAP240*-like gene, has been recently isolated and several mutations are suggested to be responsible for a subset of TGA patients [6]. Isolated mutations in *ZIC3 *[7] and *CFC1 *(human CRYPTIC gene) [8,9] have also been detected in patients with TGA. *ZIC3 *and *CFC1 *have been shown before to be involved in laterality defects in humans [8,10]. However the total number of mutations detected so far within these three genes is not sufficient to explain the high incidence of dTGA and point towards strong heterogeneity.

As cardiac neural crest cells contribute to the formation of the outflow septum that divides the common outflow tract, an association between neural crest disturbance and TGA has been suggested. Extirpation experiments in chick could show that neural crest cells contribute to normal aorticopulmonary septation. Deletion of those cells causes malformation of the aorticopulmonary septum resulting in common arterial outflow channels or transposition of the great arteries [11,12]. Pitx2, a bicoid-related homeodomain transcription factor involved in eye, heart and craniofacial development and establishment of left-right asymmetry, is expressed in several tissues of the developing mouse embryo including neural crest derived organs [13]. In humans, *PITX2 *haploinsufficiency causes Axenfeld-Rieger Syndrome (ARS), an autosomal dominant disorder involving ocular, dental and umbilical defects [14] and, in some patients with unknown mutations, also cardiac defects [15,16]. Most interestingly, Pitx2 loss of function experiments in mice cause severe cardiovascular defects including transposition of the great arteries [17-20]. Kioussi et al. reported that Pitx2^-/- ^mice, that survive up to E15, invariantly exhibit major cardiac outflow tract abnormalities, amongst which 30% show incomplete septation of the great arteries, that may develop with double outlet right ventricle (DORV) or transposition of the great arteries [20]. Deletion of the *Dvl2 *gene [21], which is regulated by the same pathway as *Pitx2*, leads to the same severe outflow tract malformations, indicating a strong implication of this pathway in the outflow tract phenotype. These lines of evidence prompted us to investigate whether *PITX2 *mutations in humans can also contribute to the etiology of TGA.

## Methods

### Human subjects and genomic DNA

Peripheral-blood samples were taken from healthy individuals and patients with simple dTGA after informed consent had been obtained, after approval by the institutional review board of ethics of the Medical Department of the University of Heidelberg and the Newcastle and North Tyneside Health Authority Joint Ethics Committee. Genomic DNA was prepared using the Puregene DNA Isolation Kit (Gentra, Inc., USA).

### PCR and mutation screening

Amplifications were performed using the High Fidelity System (Roche) according to the manufacturer's protocol. Primers were designed according to the *PITX2 *sequence gene bank accession number AF238048 and respective sequences are given in table 1. Mutation screening was performed using denaturing high performance liquid chromatography (DHPLC). A WAVE DNA-Fragment Analysis System (Transgenomic Inc., Cheshire) was used.

### Sequencing

Sequencing was performed on a MegaBACE sequencer (Amersham Bioscience, Piscataway) using the DYEnamic™ ET terminator Cycle Sequencing Kit following the manufacturer's protocol. Sequencing reactions were performed on both DNA strands. Sequences were analyzed using the Clustal program (German Cancer Research Center, Biocomputing Facility HUSAR, Heidelberg).

## Results and Discussion

**Figure 1:**
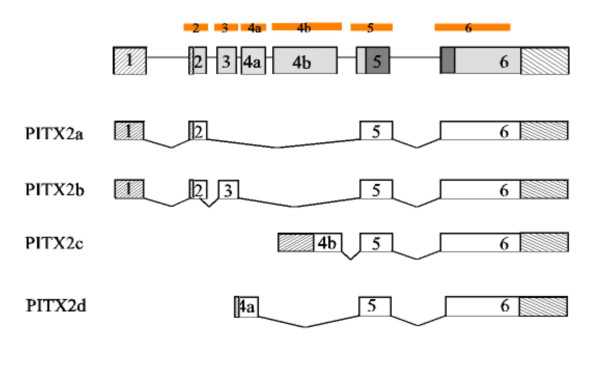
Schematic diagram of *PITX2 *isoforms a, b, c and d. The genomic organization of the *PITX2 *gene is given on top, exons are numbered, 5' and 3' UTRs of the different possible transcripts are indicated by striped boxes and the homeodomain is shaded in dark grey. Modified from Cox et al, 2002 [24]. The regions of the *PITX2 *gene included in the mutational screening are indicated as orange bars at the very top of the scheme.

96 patients with dTGA were analyzed for mutations in *PITX2 *by DHPLC and direct sequencing. All coding exons of *PITX2 *(exon 2 to 6, including both alternatively spliced exons 4a and b) were amplified by intron-specific exon-flanking primers to screen exon-intron junctions (table 1, figure 1). Non-coding regions (exon 1 and the 3'part of exon 6), intronic regions beyond the intronic sequences covered by the amplification, and promoter elements were not examined. We identified seven intronic and UTR variants, two silent mutations and two polymorphisms within the coding region. Most of these variants were found also in similar frequencies in 100 control individuals and are therefore unlikely to be of functional relevance. The missense mutation detected in exon 4b (204C>A, P65T) most likely represents a polymorphic variant compared to the sequence in the database, as the heterozygous form was invariantly detectable in all tested patients and controls. This finding also excludes large deletions in the patients affecting the whole gene locus. Three intronic (IVS2+7A>G, IVS3+11G>T, IVS4a-62C>A) and one silent mutations (30G>C Ser10Ser) were not detectable in 100 controls. One variant in the 5'UTR (2–40T>C) and one missense mutation (30C>T S27F) were only found once in control individuals (table 2).

We report on the mutation screening of *PITX2*, as we considered it to be an interesting candidate gene for TGA due to its role in regulating asymmetric cardiac morphogenesis [22] and interesting data from mouse studies. Impaired Pitx2 function in mice leads to severe cardiac malformations [17-20]. It has been suggested that altered *PITX2 *expression in the outflow tract could underlie either TGA or DORV [22].

*PITX2 *comprises three major isoforms, formed by differential splicing or alternative promotor usage: *PITX2a*, *b*, *c*, as well as one minor isoform *PITX2d *(Fig 1). We have included all coding exons in our screening as all forms exhibit a differential expression pattern [18,19]. *Pitx2c *is of special interest, as only this isoform is asymmetrically expressed within the lateral plate mesoderm and the heart and governs asymmetric organ morphogenesis in a dose-dependent manner [23,19]. Furthermore, the newly identified minor isoform, *PITX2d*, that in fact does not bind to DNA, was included in the study since it may influence expression levels of the other splice variants and also regulate the transcriptional activity of the major isoforms on protein level [24]. As only low amounts of PITX2 are required for normal cardiac development and as the different isoforms can possibly compensate for each other in some cell populations, it might require a combination of different sequence variants within different isoforms of the gene to dramatically reduce PITX2 function and therefore manifest a cardiac phenotype.

## Conclusion

To address whether altered PITX2 function could also contribute to the formation of dTGA in humans, we screened the coding regions as well as exon-intron boundaries of the *PITX2 *gene for mutations in 96 patients with dTGA. The majority of detected variants, however, were also found in controls with comparable frequency. Three intronic and one silent mutation could not be detected in 100 controls. As they were only found once in the cohort of 96 patients and as none of the variants was found within the evolutionary conserved homeodomain, we consider them to be rare polymorphisms rather than functional mutations, although we cannot totally exclude the latter possibility. Further investigations will have to evaluate whether these sequence variants might change splicing processes. Due to the study design we can also not exclude mutations in the very 5'and 3' UTRs and within introns as well as the promoter regions of the gene. Nevertheless, we conclude that the detected mutations in *PITX2 *are not a common cause of dTGA.

## Competing interests

The author(s) declare that they have no competing interests.

## Authors' contributions

NM designed the study, BN participated in the mutation analysis and drafted the manuscript, RR and KS performed PCRs and sequencing reactions. HJR, JG and EG provided patient care and collected blood samples. GR was involved in study design and supervision and finalized the manuscript. All authors read and approved the final manuscript.

## Pre-publication history

The pre-publication history for this paper can be accessed here:



## Figures and Tables

**Table 1:** Primer pairs used for mutation analysis, covering the coding region of *PITX2*.

**exon**	**primer name**	**sequence 5'> 3'**	**T_A_°C**	**reference**
2	PITX2-exon2for: PITX2-exon2rev:	tag tct cat ctg agc cct gc gcg att tgg ttc tga ttt cct	60	Ref: [25] this paper
3	PITX2-exon3bfor: PITX2-exon3brev:	ttg ctc ttt gtc cct ctt tct cct cgg agt gtc taa gtt caa gca gca	60	this paper
4a	PITX2-exon4afor: PITX2-exon4arev:	ccg cct ctg gtt tta aga tg gca aag acc ccc ttc ttc tc	60	this paper
4b	PITX2-exon4bfor: PITX2-exon4brev:	ctt gac act tct ctg tca gg aag cgg gaa tgt ctg cag g	60/56/52*	Ref: [25]
5	PITX2-exon5for: PITX2-exon5rev:	cag ctc ttc cac ggc ttc t ttc tct cct ggt cta ctt gg	60	Ref: [25]
6	PITX2-exon6for: PITX2-exon6rev:	gta atc tgc act gtg gca tc agt ctt tca agg gcg gag tt	65	Ref: [25]

T_A_: Annealing temperature

* step down PCR was performed with three temperatures for 10/10/15 cycles.

**Table 2:** Summary of *PITX2 *sequence variations in the dTGA study cohort

**patients (n = 96)**			**controls**	
**type of variation:**	**specific variation**	**variant frequency (%)**	**number of controls**	**frequency (%)**

**intronic/UTR variations:**	2–40T>C (5'UTR exon 2) 2–18T>C (5'UTR exon 2) IVS2+7A>G (intron 2) IVS2-106C>A (intron 2) IVS3+11G>T (intron 3) IVS4a+11G (intron 4a) IVS4a-62C>A (intron 4a)	10 (10.4%) 0 (0%) 1 (1.04%) 17 (17.7%) 1 (1.04%) 30 (31,25%) 1 (1.04%)	100 100 100 100 100 100 100	12 (12%) 1 (1%) 0 (0%) 20 (20%) 0 (0%) 39 (39%) 0 (0%)

**silent mutations:**	30G>C (S10S) (exon 2) 63C>T (A21A) (exon 4b)	1 (1.04%) 1 (1.04%)	100 100	0 (0%) 2 (2%)

**polymorphism within coding region:**	30C>T (S27F) (exon 3) 204C>A (P65T) (exon 4b)	0 (0%) 96 (100%)	100 100	1 (1%) 100 (100%)

UTR: untranslated region
